# The relationship between motor and cognitive performance and cortical activity in patients with multiple sclerosis: a systematic review of functional near-infrared spectroscopy studies

**DOI:** 10.3389/fresc.2026.1851559

**Published:** 2026-07-15

**Authors:** Hongjiao Sun, Haonan Xing, Kechun Hu, Zhongyu Wu, Jiawei Wu

**Affiliations:** 1 Department of Inpatient and Medical Record Management, Air Force Medical Center, Air Force Medical University, PLA, Beijing, China; 2 Rehabilitation Hospital Affiliated to National Research Center for Rehabilitation Technical Aids, Beijing, China

**Keywords:** brain, dual-task paradigm, fNIRS, motor-cognitive performance, multiple sclerosis

## Abstract

**Background:**

This review aimed to examine the relationship between motor/cognitive performance and cortical activity in individuals with multiple sclerosis (MS) under single- and dual-task conditions using functional near-infrared spectroscopy (fNIRS).

**Methods:**

The review was preregistered with PROSPERO (CRD420261402862) and followed the PRISMA protocol. PubMed, Cochrane Library, and Web of Science were systematically searched, and 18 relevant studies were included to address four core questions: (1) whether cortical activation differs between MS patients and healthy controls; (2) how activation patterns vary under single- vs. dual-task conditions; (3) whether activation is associated with motor/cognitive performance; and (4) the potential mechanisms underlying altered cortical activation.

**Results:**

The findings suggest that some MS patients may exhibit increased cortical activation alongside reduced performance. During single tasks, activation in motor/cognitive regions was often higher than that observed in healthy controls, and in some studies, this activation correlated with the severity of disability. This pattern became more pronounced under dual-task conditions, with concurrent declines in gait and cognitive performance reported. Rehabilitative interventions may help modulate such activation.

**Conclusions:**

Certain MS patients demonstrate increased task-related cortical activation accompanied by reduced performance. While this may reflect alterations in neural efficiency or neurovascular coupling, it could also indicate compensatory recruitment or increased attentional effort, depending on the task, disability severity, and disease-related factors. fNIRS may serve as a useful monitoring tool, and future multimodal EEG-fNIRS research is warranted to clarify the underlying mechanisms.

**Systematic Review Registration:**

https://www.crd.york.ac.uk/prospero/, identifier CRD420261402862.

## 1 Introduction

Multiple sclerosis (MS) is a neurodegenerative disease characterized by inflammation, demyelination, and axonal degeneration, which leads toa range of neurological deficits and significantly impairs patients' quality of life (1). Studies have shown that 80% of MS patients experience motor impairment, while 40%–70% present with concomitant cognitive impairment (e.g., declines in executive function and working memory), and the concurrent impairment of motor and cognitive coordination is is therefore considered one of the most representative comorbid features of MS patients (2–4). Further evidence suggests that motor and cognitive functions are interrelated or coupled in both aging and multiple sclerosis (5). Currently, approximately 2.8 million people worldwide are living with MS, with a prevalence of 35.9 cases per 100,000 population (6). As the high incidence of MS increasingly shifts toward the elderly population, the synergistic decline in these two domains is closely associated with disease severity, disability progression, and prognosis in MS patients, thereby posing significant challenges for the evaluation and treatment of this population (7).

Motor and cognitive performance is particularly critical for patients with MS, especially in daily life scenarios (e.g., walking while conversing). Daily activities typically involve the integration of cognitive and motor tasks, a process commonly referred to as dual-task processing (8). During this process, the functional specialization and network integration of the cerebral cortex collectively determine the level of motor and cognitive performance (9). Emerging evidence indicates that MS patients experience difficulties in cognitive-motor integration, which may be attributed to abnormal neurovascular coupling (NVC) and altered metabolic cortical activation, resulting in an inability to effectively allocate attentional resources. Consequently, ccompared with single-task conditions (motor or cognitive alone), MS patients exhibit declines in both motor performance (e.g., reduced gait speed, impaired balance) and cognitive performance under dual-task conditions (10–13). Therefore, elucidating the changes in motor/cognitive performance and their relationship with cortical activity in MS patients will help clarify the mechanisms underlying motor-cognitive impairment and inform the development of effective rehabilitation strategies for this population.

Traditional neuroimaging techniques (e.g., fMRI) are susceptible to motion artifacts, making it difficult to capture cortical activity in patients during real-world motor tasks (14). While portable electroencephalography (EEG) can monitor neural electrical activity in real time, it has limited spatial resolution and cannot accurately localize the key brain regions involved in motor planning, execution, and regulation (15). Based on the principles of NVC and optical spectroscopy, increased cerebral neural activity leads to elevated cerebral metabolic demand, resulting in decreased deoxygenated hemoglobin (HbR) levels and increased oxygenated hemoglobin (HbO_2_) levels (16, 17). Functional near-infrared spectroscopy (fNIRS) is a non-invasive, neuroimaging tool with moderate spatial resolution and favorable cost-effectiveness. It quantifies cerebral hemodynamic variations of oxy/deoxyhemoglobin to reflect brain activity and has been widely applied in functional neuroscience research (18–20). Previous studies have shown that fNIRS can be used to evaluate motor or cognitive performance in MS patients under single or dual-task conditions, with a particular focusing on monitoring activity in brain regions closely involved in motor planning, execution, and cognitive control. These regions include the prefrontal cortex (PFC), primary motor cortex (M1), premotor cortex (PMC), supplementary motor area (SMA), premotor area (PM), sensorimotor cortex (SMC), and frontopolar area (FPA) (21, 22). Therefore, fNIRS provides an ideal tool for analyzing the neural mechanisms underlying motor-cognitive impairment in MS patients.

In conclusion, this systematic review aims to integrate existing evidence from fNIRS studies and address the following four core questions:(1) whether cortical activation differs between MS patients and healthy controls; (2) how activation patterns vary under single- vs. dual-task conditions; (3) whether activation is associated with motor/cognitive performance; and (4) the potential mechanisms underlying altered cortical activation.

## 2 Materials and methods

### 2.1 Study selection criteria

This systematic review was preregistered with PROSPERO (CRD420261402862) and followed the Preferred Reporting Items for Systematic Reviews and Meta-Analyses protocol. We established the inclusion criteria based on the PICOS framework. Studies meeting the following criteria were included: (1) Population: Adults aged ≥18 years with a confirmed diagnosis of MS; (2) Intervention: Any single/dual-task paradigm combining cognitive tasks or motor tasks (including single cognitive tasks, single motor tasks, or dual tasks) administered by researchers; (3) Comparison: Control groups performing single cognitive tasks, single motor tasks, or dual tasks combining cognitive and motor tasks; (4) Outcome: Studies that evaluated cerebral cortical hemodynamic responses during single or dual tasks using fNIRS; (5) Study design: Cross-sectional studies, randomized controlled trials (RCT), cohort studies, and pre-post studies, etc. Exclusion criteria: (1) Studies not employing fNIRS; (2) Non-original articles, such as reviews, editorials, commentaries, study protocols, conference abstracts, and letters; (3) Studies restricted to healthy individuals, patients with other diseases, or animals; (4) Studies using other cortical activation detection modalities, such as EEG, functional magnetic resonance imaging (fMRI), or transcranial magnetic stimulation (TMS); (5)Non-English article.

### 2.2 Search strategy

We conducted systematic searches in three electronic databases: PubMed, Web of Science, and Cochrane Library, with the search cutoff date set to December 31, 2025. The search strategy comprised all possible combinations of the following three groups of keywords and their corresponding Medical Subject Headings (MeSH) terms: (1) Multiple sclerosis-related terms: multiple sclerosis, MS, adults, older adults/elderly; (2) Task paradigm-related terms: dual-task, cognitive-motor performance, gait, walking, cognition, executive function, memory, motor function, mobility; (3) fNIRS-related terms: fNIRS, functional near-infrared spectroscopy, near-infrared spectroscopy. Manual supplementary searches were also conducted in Embase and Scopus.

Study eligibility was determined by screening the titles, abstracts, and full texts of the retrieved articles. 2 independent reviewers (WJW and SHJ) screened the titles, abstracts, and full texts to assess eligibility. Any discrepancies were resolved through discussion.

### 2.3 Data extraction

The following details were extracted from each included study: title, publication year, authors; characteristics of MS patients (sample size, age, disease subtype); fNIRS techniques used to measure brain activity (device type, wavelength, regions of interest, motion artifacts, extracerebral contamination, short-separation regression, preprocessing variability, physiological noise correction); task type and paradigm (i.e., single-task, dual-task); intervention duration, task performance, and cortical activation outcomes, including statistical correlations, gait characteristics, cognitive performance, etc.

### 2.4 Risk of bias in individual studies

2 independent reviewers (WJW and SHJ) assessed the quality of each included study using the research quality assessment tools from the US National Institutes of Health (NIH) (23). Cross-sectional studies were evaluated using the Quality Assessment Tool for Observational Cohort and Cross-Sectional Studies, RCT were assessed with the Quality Assessment of Controlled Intervention Studies, and case-control studies were evaluated using the Quality Assessment Tool for Case-Control Studies. The quality assessment was performed to measure the strength of scientific evidence but was not used to determine study eligibility.

## 3 Results

### 3.1 Overview of included studies

**Figure 1:**
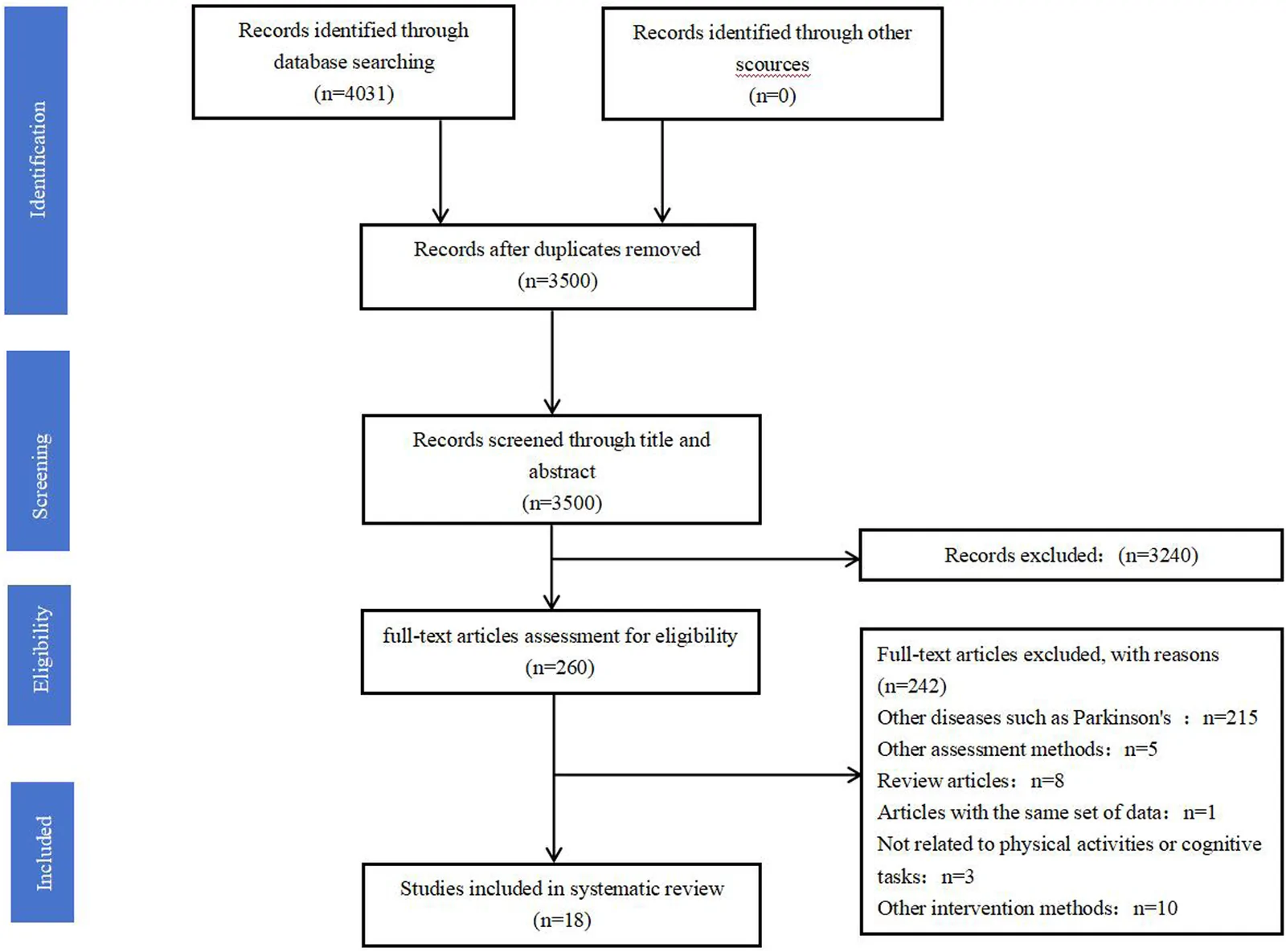
Search and screening process.

As shown in Figure 1, a total of 4,031 studies were identified through keyword searches. Among them, 531 studies were removed due to duplication, 3,240 studies were excluded during title and abstract screening, and 260 studies underwent full-text review. Of these 260 studies, 242 were excluded for failing to meet the above screening criteria. The remaining 18 studies were included in this review (Figure 1).

### 3.2 Basic characteristics of included studies

A total of 572 patients with MS and 291 healthy controls (HC) were enrolled across the included studies. Among the MS patients, 140 had relapsing-remitting MS, 60 had progressive MS, and the remaining patients were not subtyped. The mean age of MS patients ranged from 29 to 69 years, while that of HC ranged from 30 to 75 years. Among all included studies, 1 was a RCT, 2 were case-control studies, and the rest were cross-sectional studies. Cognitive tasks included reciting alternate letters, arithmetic task, serial 7^，^s subtraction, memory tasks, etc. Motor tasks included normal walking, stepping over known/unknown obstacles, 6-minute normal walking on a treadmill, dishwashing tasks in virtual reality (VR) and real-world settings, assisted walking, and other related tasks (Table 1). When investigating the activation patterns of MS patients across different tasks, different cortical regions were taken into consideration. The results indicated that the PFC was the most frequently studied area across the included studies (*n* = 12). In contrast, only a small number of studies (*n* = 6) examined hemodynamic responses in multiple cortical regions, including motor-related areas such as the SMA, PMC, FPA, SMC, primary motor cortex (MC), and the entire cerebral cortex (Table 2).

**Table 1:** Summary of motor and cognitive performance and cortical activation outcomes in studies of multiple sclerosis.

Authors year	Participant characteristics	Age	Task paradigms	Motor and cognitive performance and cortical activation results
Hernandez ME et al., 2016 (29)	8 MS (F:6, M:2)	57 ± 5	ST: normal walking	↓Step speed, ↓correct letters and ↑HbO_2_ level in PFC under DT condition compared with HC or ST condition in MS group.
	7 HC (F:6, M:2)	61 ± 4	DT: ST + reciting alternate letters	
Chaparro G et al., 2017 (31)	10 MS (F:8, M:2)	56.2 ± 5.1	ST: walking on a treadmill with no or PBWS	↓Step speed and ↑HbO_2_ level in PFC across all tasks and conditions in the MS compared with HC.
	12 HC (F:9, M:3)	63.1 ± 4.4	DT: ST + reciting alternate letters	↓Cognitive performance under DT condition in the both group compared with ST (PBWS).
Borragán G et al., 2018 (25)	10 Relapsing-remitting MS (F:40, M:13)	42.1 ± 7.1	ST：Tload Dback with HCL or LCL.	↓HbO_2_ level in DLPFC,↑cognitive fatigue in the MS group.
	11 HC (F:14, M:14)	34.18 ± 4.64		
Saleh S et al., 2018 (10)	14 Relapsing-remitting MS (F:12, M:2) 14 HC (F/M -)	50 ± 8	ST1: serial 7’s subtraction cognitive task	↑rPMC, ↑rSMA and↓correct letters under DT condition compared with ST1.
		50 ± 9	ST2: normal walking	A correlation between ↓ step speed and ↑ SMA during DT condition in the MS group.
			DT: ST1 + ST2	
Hernandez ME et al., 2019 (24)	10 MS (F:8, M:2)	56.2 ± 5.1	ST1: recite alternate letters of the alphabet backward while standing still 30s	↑HbO_2_ level in PFC under ST1 or ST2 condition compared with HC in MS group.
	12 HC (F:9, M:3)	63.1 ± 4.4	ST2: normal walk on the treadmill within the 10-cm width of a virtual beam	←Step speed, and ↑HbO_2_ level in PFC across the DT trials compared with ST2, but compared with HC, smaller increases in the MS.
			DT: ST1 + ST2	↓Cognitive performance under DT condition compare with ST1 in the both group.
Broscheid KC et al., 2020 (27)	16 MS (F:14, M:2)	41 ± 12	ST: 6-min walk test (6MWT)	↑HbO_2_ level in DLPFC and FPC under ST condition compared with HC group.
	19 HC (F:15, M:4)	42.2 ± 9.8		
Lamberti N et al., 2021 (32)	12 RAGT MS (F:7, M:5)	56 ± 10	ST: walking on a treadmill	Baseline, ↓Step speed and ↑cortical activity under the ST condition in the MS group compared with HC.
	12 OW MS (F:4, M:8)	57 ± 11	RAGT: walking in the Lokomat treadmill	After recovery, ↑cortical activity in the OW group,↓cortical activity in the RAGT.
	5 HC (F/M:-)	–	OW: Physiotherapist-assisted overground walking	
			Time/Frequency: 40 min/2/week, 6 weeks	
de Aratanha MA, 2022 (30)	20 MS (F/M:-)	35.3 ± 6.3	ST1: normal walking	↑HbO2 level in PFC and PMC under all tasks in MS group compared with HC,and as the task difficulty increases, the activation level also increases accordingly.
	19 HC（F/M:-）	35.5 ± 8	ST2: ST1 + n-back task	Compared with ST1, ↑HbO2 level in PFC and PMC under ST2 in both groups.
Broscheid KC et al., 2022 (35)	20 MS (F:13, M:7)；	48.3 ± 9	DT: 6MWT on the treadmill + arithmetic task	←HbO_2_ and ←PFC activity during walking under DT condition compared with baseline condition in the MS and HC group.
	24 HC (F:17, M:7)	48.6 ± 7.9		↓Step speed and ↓cognitive performance (increased error rate)under DT condition in the MS group.
Kupchenko Y et al., 2023 (33)	18 MS (F:12, M:6)	36.1 ± 11.7	ST1: normal forward walking	↓Step speed and ↑HbO_2_ level in PFC (DLPFC/FPC/FEF) under the DT1 condition compare with ST1 in the both groups, but more pronounced in HC group.
	17 HC (F:13, M:4)	37.5 ± 13.8	ST2: normal backward walking	↑HbO_2_ level in DLPFC/FEF in the MS group, and in FPC/FEF in the HC group under ST2 condition compare with ST1.
			DT1: ST1 + serial-7 backward	
			DT2: ST2 + serial-7 backward	
Al-Shargie F et al., 2024 (39)	9 MS (F:7, M:2)	51.5 ± 8.0	ST1: walking with predictable obstacles on the treadmill	↑PMC, ↑MC, and ↑SAC, under ST1 or ST2 condition in the MS group compared with HC group.
	5 HC (F:4, M:1)	51.2 ± 6.4	ST2: walking with non-predictable obstacles on the treadmill	Compare with HC, MS exhibited more widespread reductions in cortical activations during non-predictable obstacle avoidance.
Baldasso BD et al., 2024 (38)	15 MS (F:12, M：3)	43.8 ± 8.87	ST1: serial 7′s subtraction cognitive task	↓PFC with the complexity of cognitive tasks increases (From ST1 to ST2) in the MS group.
	12HC (F:10, M:2)	45.2 ± 10.52	ST2: ST1 + alternate answers with letters of the alphabet in ascending order	
Hernandez ME et al., 2024 (26)	51 Lower Disability MS (F:36, M:15)	65.1 ± 4.2	ST1: normal walking	↑HbO_2_ and ↑PFC under ST1 condition in the Higher Disability MS group, compared with Lower Disability MS group.
	48 Higher Disability MS (F:30, M:18)	64.5 ± 5.1	ST2: reciting alternate letters	↑PFC from ST1 to a cognitively-demanding task (ST2 or DT) in the Higher Disability MS group, compared with Lower Disability MS group.
			DT: ST1 + ST2	↓Step speed and ↓correct letter generation rate under DT condition in the Higher Disability MS group, compared with Lower Disability MS group.
Holtzer R et al., 2024 (34)	94 MS (F:65, M:29)	64.76 ± 4.19	ST1: normal walking	↑HbO_2_, ↑PFC ,↓Step speed, and ↓correct letter generation rate under DT condition in the both group compared with ST1 or ST2.
	104 HC (F:67, M:37)	68.19 ± 7.1	ST2: reciting alternate letters	
			DT: ST1 + ST2	
Holtzer R et al., 2024 (36)	53 Relapsing-remitting MS (F:40, M:13)	65.02 ± 4.17	ST1: normal walking	↓Correct letters and ↑HbO_2_ level in PFC under DT condition compared with ST1 or ST2.
	28 Progressive MS (F:14, M:14)	64.64 ± 4.31	ST2: reciting alternate letters	↑Step speed and ↓falling under ST1 condition in the Progressive MS group.
			DT: ST1 + ST2	↑HbO_2_ level in PFC were associated with and↓falling of relapsing-remitting MS group.
Santinelli FB et al., 2024 (28)	15 MS (F/M:-)	42 ± 11	ST1: normal walking	↓Walking distance and ↑FPA under the ST1 condition in the MS compared with HC.
	16 HC (F/M:-)	45.2 ± 13.2	ST2: serial 7′s subtraction cognitive task while standing still	↓Correct letters MS showed worse performance in the ST1, ST2, and DT compared to HC.
			DT: ST1 + ST2	HC ↑FPA in the ST2 and DT, Conversely, MS maintained similar cortical activity.
Hernandez ME et al., 2025 (37)	63 Relapsing-remitting MS (F:47, M:16)	64.7 ± 4.2	ST1: normal walking	↓Step speed, ↓correct letter generation rate, ↑HbO_2_ and ↑PFC under DT condition in the progressive MS group, compared with relapsing-remitting MS group.
	32 Progressive MS (F:16, M:16)	65.1 ± 5.6	ST2: reciting alternate letters	
			DT: ST1 + ST2	
Lavi R et al., 2025 (40)	14 MS (F:8, M:6)	50.9 ± 5.1	ST1: VR (wash the dishes)	↓PMC and ↓SMA under ST1 and ST3 condition in the MS group compared with HC group.
	14 HC (F:5, M:9)	47.6 ± 11.6	ST2: VR (wash the dishes + Memory task)	In the ST2, ↑SMA, ↑PMC, and HbO_2_ in the MS group compared with HC group.
			ST3: AP (wash the dishes)	←Cortical activation across differences task conditions in the MS group.
			ST4: AR (wash the dishes + Memory task)	

MS, multiple sclerosis; HC, healthy people; RAGT, robot-assisted gait training; OW, overground walking; Age, Mean ± SD; PBWS, partial body weight support; HCL, high cognitive load; LCL, low cognitive load; 6MWT, 6-minute walk test; PFC, prefrontal cortex; DLPFC, dorsolateral prefrontal cortex; SMA, supplementary motor area; PMC, premotor cortex; FPA, frontopolar area; FEF, frontal eye field; SAC, somatosensory association cortex; DT, dual-task; VR, virtual reality; ↑, increase significantly; ↓, decrease significantly; ←, no significant differences.

**Table 2a:** Summary of fnirs parameters.

Authors year	Type	Scalp location method	fNIRS measures	Wavelength (nm)	Sensors'distance (cm)	Sampling frequency (Hz)	Focus area
Hernandez ME et al., 2016 (29)	fNIRS Devices, LLC, Potomac, MD	10–20 system	HbO_2_	730/850	2.5	2	PFC
Chaparro G et al., 2017 (31)	fNIR Imager 1000 (fNIR Devices LLC, Potomac, MD)	10–20 system	HbO_2_	—	2.5	2	PFC
Borragán G et al., 2018 (25)	fNIRS (BrainSight, V2.3b16, NIRS, Rogue Research Inc., Canada)	10–20 system	HbO_2_	685/830	3	20	DLPFC
Saleh S et al., 2018 (10)	NIRSport™, NIRX, Germany	10–5 system	HbO_2_	760/850	—	6.25	PMC/SMA
Hernandez ME et al., 2019 (24)	—	10–20 system	HbO_2_	730/850	2.5	2	PFC
Broscheid KC et al., 2020 (27)	NIRSport (NIRx Medical Technologies, NY, USA)	10–20 system	HbO_2_	760/850	3–4	7.81	DLPFC/FPA
Lamberti N et al., 2021 (32)	NIRScout, NIRx Medical Technologies LLC, Glen Head, NY, USA	10–20 system	HbO_2_	760/850	3	3.46	PMC/SMC
de Aratanha, MA (30)	NIRSport, NIRx Medizintechnik GmbH, Germany	10–20 system	HbO_2_	730/860	—	7.8	PFC/DLPFC/PMC/SMA
Broscheid KC et al., 2022 (35)	NIRSport (NIRx Medical Technologies, New York, USA)	10–20 system	HbO_2_	760/850	3–4	7.81	PFC
Kupchenko Y et al., 2023 (33)	NIRSport, NIRx Medical Technologies, NY, USA	10–20 system	HbO2	760/850	3–4	7.81	PFC/DLPFC/FPC/FEF
Al-Shargie F et al, 2024 (39)	NIRSport2 system (tandem NIRSport 2; LLC NIRx Medical Technologies)	10–20 system	HbO_2_	760/850	3	7.81	PMC/MC/SAC
Baldasso BD et al., 2024 (38)	NIRScoutX® 16 × 16 imaging system (NIRx Medical Technologies, Berlin, Germany)	10–20 system	HbO_2_	—	3	3.9	PFC/DLPFC
Hernandez ME et al., 2024 (26)	fNIRS Imager 1,100 (fNIRS Devices, LLC, Potomac, MD)	10–20 system	HbO_2_	730/850	2.5	2	PFC
Holtzer R et al., 2024 (34)	fNIRS Imager 1,100 (fNIRS Devices, LLC, Potomac, MD)	10–20 system	HbO_2_	730/850	2.5	2	PFC
Holtzer R et al., 2024 (36)	fNIRS Imager 1,100 (fNIRS Devices, LLC, Potomac, MD)	10–20 system	HbO_2_	760/850	2.5	2	PFC
Santinelli FB et al., 2024 (28)	NIRSport2, NIRx Medical Technologies, Germany	10–10 system	HbO_2_	760/850	3–4	10.2	FPA/DLPFC
Hernandez ME et al., 2025 (37)	fNIRS Imager 1,100 (NIRS Devices, LLC, Potomac, MD)	10–20 system	HbO_2_	730/850	2.5	2	PFC
Lavi R et al., 2025 (40)	NIRSport2, NIRx Medical Technologies, Berlin, Germany	10–20 system	HbO_2_	760/850	3–4	7.81	PMC/SMA/SAC

PFC, prefrontal cortex; DLPFC, dorsolateral prefrontal cortex; SMA, supplementary motor area; PMC, premotor cortex; FPA, frontopolar area; FEF, frontal eye field; SAC, somatosensory association cortex; —, indeterminacy.

**Table 2b:** Summary of fnirs parameters.

Authors year	Preprocessing pipelines	Motion correction methods	Short-separation channel	Statistical correction procedures
Hernandez ME et al., 2016 (29)	Blinded data analysis: saturation/dark current removal, 0.14 Hz low-pass filtering, MBLL conversion, 10s baseline correction	Visual inspection for saturation/dark current	—	10-second baseline correction with zero-mean adjustment and normalization to calculate relative HbO₂ changes
Chaparro G et al., 2017 (31)	MATLAB R2014a: Visual noise inspection, 0.14 Hz low-pass filtering, MBLL conversion, 30s trial analysis (first 10s vs. last 10s)	Visual noise inspection + Low-pass filtering	—	Linear mixed models + post-hoct-tests with Hochberg's step-up correction for multiple comparisons
Borragán G et al., 2018 (25)	Homer2: automated noisy channel removal, 0.009–0.08 Hz low-pass filtering, MBLL conversion, double normalization (AUs + corrected baseline)	Automated noisy channel removal via correlation analysis	—	Tukey HSD test was employed for *post hoc* corrections
Saleh S et al., 2018 (10)	NIRSlab Matlab: channel rejection (CV > 15%), 0.01–0.14 Hz bandpass filtering, MBLL conversion, 15s baseline correction, trial averaging	Discontinuities and spike artifacts removal	—	the index of hemoglobin differential (HbO_2_ – HbR) calculated for activation analysis
Hernandez ME et al., 2019 (24)	Blinded data analysis: saturation/dark current removal, 0.14 Hz low-pass filtering, MBLL conversion	Visual inspection for saturation/ dark current	—	Task-related changes calculated by comparing trial averages to pre-task baseline values
Broscheid KC et al., 2020 (27)	Homer2: channel pruning (SNR = 2), 0.5 Hz low-pass filtering, MBLL conversion, GLM-HRF modeling (−10–45s window, 5–25s analysis interval)	Spline interpolation + Savitzky- Golay filter	The nearest Short-separation channel regression	GLM + OLS + HRF estimation
Lamberti N et al., 2021 (32)	NIRSlab software: quality check, bandpass filtering, exclusion of channels with poor SNR or gain ≥7, MBLL conversion	Removal of discontinuities, spikes, and movement artifacts during preprocessing	—	Analysis focuses on area under the curve quantification of oxy-Hb
de Aratanha MA, 2022 (30)	BrainAnalyzIR toolbox: Hemoglobin concentrations calculated using the modified Beer-Lambert law (age and wavelength adjusted DPF), signal pre-whitening using the AR-IRLS algorithm, GLM	AR-IRLS pre-whitening algorithm	—	Multiple comparison correction using the Benjamin-Hochberg algorithm
Broscheid KC et al., 2022 (35)	Homer3: channel pruning (signal intensity & SD-based), 0.01–0.09 Hz bandpass filtering, MBLL conversion, GLM + HRF modeling, 30–55 s time window	Spline interpolation + Savitzky- Golay filter	The nearest Short-separation channel regression	GLM + OLS + HRF estimation
Kupchenko Y et al., 2023 (33)	Homer3:channel pruning (SNR = 2), 0.01–0.09 Hz bandpass filtering, MBLL conversion (age-adjusted DPF), GLM + HRF modeling, 3rd-order polynomial drift correction	Spline interpolation + Savitzky – Golay filter	The nearest Short-separation channel regression	GLM + OLS
Al-Shargie F et al., 2024 (39)	Brain AnalyzIR toolbox: quality check, 2nd-order detrending, 0.01–0.2 Hz bandpass filtering, 30s baseline correction, 13-minute data window	Temporal Derivative Distribution Repair + PCA algorithms	—	PSD and the directed FCN analyses
Baldasso BD et al., 2024 (38)	Homer3: channel pruning (SN ＜ 8), 0.01–0.09 Hz bandpass filtering, MBLL conversion, GLM + HRF modeling	spline + Wavelet methods	Short-separation channel regression (source-detector distance <15 mm)	GLM + OLS
Hernandez ME et al., 2024 (26)	Daubechies 5 wavelet denoising, spline filtering + 0.08 Hz low-pass, MBLL conversion (age and wavelength adjusted DPF), 10-second baseline correction	spline filtering	—	Changes corrected relative HbO2 changes calculated relative to 10-second baselines
Holtzer R et al., 2024 (34)	Daubechies 5 wavelet denoising, spline filtering + 0.08 Hz low-pass, MBLL conversion (age and wavelength adjusted DPF), 10-second baseline correction	spline filtering	—	Changes corrected relative HbO_2_ changes calculated relative to 10-second baselines
Holtzer R et al., 2024 (36)	Daubechies 5 wavelet denoising, spline filtering + 0.08 Hz low-pass, MBLL conversion (age and wavelength adjusted DPF), 10-second baseline correction	spline filtering	—	Corrected relative HbO_2_ changes calculated relative to 10-second baselines
Santinelli FB et al., 2024 (28)	Homer2: visual channel inspection, 0.01–0.09 Hz bandpass + 0.06–0.14 Hz bandstop filtering, MBLL conversion, 7.5s baseline correction	Wavelet filter correction	The nearest Short-separation channel regression	Relative HbO₂/HbR changes calculated via baseline subtraction
Hernandez ME et al., 2025 (37)	Daubechies 5 wavelet denoising, spline filtering + 0.08 Hz low-pass, MBLL conversion (age and wavelength adjusted DPF), 10-second baseline correction	spline filtering	—	Changes corrected relative HbO₂ changes calculated relative to 10-second baselines
Lavi R et al., 2025 (40)	Homer3: channel pruning (SNR = 2), 0.01–0.09 Hz bandpass filtering, MBLL conversion (age-adjusted DPF), GLM + HRF modeling, 3rd-order polynomial drift correction	Spline interpolation + Savitzky – Golay filter	The nearest Short-separation channel regression	GLM + OLS

AU, absorption unities; CV, coefficient of variation; SNR, signal-to-noise ratio; GLM, general linear model, HRF, hemodynamic response function, OLS, ordinary least squared, DPF, differential pathlength factor; PSD, power spectrum density, FCN, functional connectivity networks, AR-IRLS, autoregressive model with iteratively reweighted least squares, –indeterminacy.

### 3.3 Methodological quality of included studies

The methodological quality of included studies was assessed using the National Institutes of Health (NIH) Study Quality Assessment Tools. 12 studies were rated as “good” and 6 as “fair”. Overall, several limitations were identified across studies: some failed to report an adequate sample size, did not provide sufficient duration for study procedures, and did not control for confounding variables in their analyses. However, task types and paradigms were described in detail across all studies, and the tasks performed by participants were comparable (Table 3).

**Table 3:** Quality assessment.

Authors year	Q1	Q2	Q3	Q4	Q5	Q6	Q7	Q8	Q9	Q10	Q11	Q12	Q13	Q14	% Score	Overall rating
Quality assessment for observational cohort and cross-sectional studies																
Hernandez ME et al., 2016 (29)	1	1	CD	1	0	0	0	1	1	NA	1	NA	NA	1	7/10 = 70%	Good
Chaparro G et al., 2017 (31)	1	0	CD	1	0	0	0	1	1	NA	1	NA	NA	1	6/10 = 60%	Good
Borragán G et al., 2018 (25)	1	0	CD	CD	0	0	0	1	1	NA	1	NA	NA	1	5/11 = 45.5%	Fair
Saleh S et al., 2018 (10)	1	0	CD	1	0	0	0	1	1	NA	1	NA	NA	0	6/11 = 54.5%	Good
Hernandez ME et al., 2019 (24)	1	0	CD	1	0	0	0	1	1	NA	1	NA	NA	1	5/10 = 50%	Fair
Broscheid KC et al., 2020 (27)	1	0	CD	1	0	0	0	0	1	NA	1	NA	NA	1	5/11 = 54.5%	Fair
de Aratanha MA, 2022 (30)	1	1	CD	1	0	0	0	1	1	NA	1	NA	NA	1	7/10 = 70%	Good
Broscheid KC et al., 2022 (35)	1	1	CD	1	0	0	0	0	1	NA	1	NA	NA	0	6/11 = 54.5%	Fair
Al-Shargie F et al., 2024 (39)	1	0	CD	CD	0	0	0	1	1	NA	1	NA	NA	0	4/10 = 40%	Fair
Baldasso BD et al., 2024 (38)	1	1	CD	1	0	0	0	1	1	NA	1	NA	NA	0	6/10 = 60%	Good
Hernandez ME et al., 2024 (26)	1	1	CD	1	0	0	0	1	1	NA	1	NA	NA	1	7/11 = 63.6%	Good
Holtzer R et al., 2024 (34)	1	1	CD	1	0	0	0	1	1	NA	1	NA	NA	1	7/11 = 63.6%	Good
Holtzer R et al., 2024 (36)	1	1	CD	1	0	0	0	1	1	NA	1	NA	NA	1	7/11 = 63.6%	Good
Santinelli FB et al., 2024 (28)	1	1	CD	1	0	0	0	1	1	NA	1	NA	NA	0	6/11 = 54.5%	Fair
Hernandez ME et al., 2025 (37)	1	1	CD	1	0	0	0	1	1	NA	1	NA	NA	1	7/11 = 63.6%	Good
Quality Assessment of Case-Control Studies																
Kupchenko Y et al., 2023 (33)	1	1	1	0	1	1	CD	CD	CD	NA	1	NA	0	NA	6/8 = 75%	Good
Lavi R et al., 2025 (40)	1	1	1	0	1	1	CD	CD	CD	NA	1	NA	1	NA	7/8 = 87.5%	Good
Quality Assessment of Controlled Intervention Studies																
Lamberti N et al., 2021 (32)	1	1	1	0	1	1	1	1	1	CD	1	0	1	1	11/13 = 84.5%	Good

Questions were assessed based on the NIH Study Quality Assessment Tools (https://www.nhlbi.nih.gov/health-topics/study-quality-assessment-tools). CD, cannot determine; NA, not applicable; 1, Yes, for the given question; 0, No, for the given question.

### 3.4 Association between task performance and cortical activation under single-task conditions

#### 3.4.1 Single cognitive tasks

3 studies reported cortical activation in patients with MS during single cognitive tasks. Compared with HC, MS patients exhibited higher PFC activation levels under single cognitive task conditions (24). A linear regression study found that reduced activation in the dorsolateral prefrontal cortex (DLPFC) was negatively correlated with increased levels of cognitive fatigue (CF) in MS patients, relative to healthy individuals (25). Within the MS patient population, higher disability severity was associated with stronger PFC activation (26).

#### 3.4.2 Single motor tasks

5 studies demonstrated that during single walking tasks, oxygenation levels in the PFC, DLPFC, PMC, frontal pole cortex (FPC), and FPA were higher in patients with MS compared with healthy controls (24, 27–30). 1 study also confirmed that PFC cortical activation levels were higher in MS patients than in healthy individuals, regardless of whether body weight support was provided (31). Another RCT using a treadmill walking task assessed the M1 and PM by calculating the hemispheric oxygenated hemoglobin area. At baseline, cortical activation was higher in MS patients than in HC. After 4 weeks of rehabilitation training, cortical activation decreased in the robot-assisted gait training (RAGT) group but increased in the observation group (OW) (32). Compared with forward walking conditions, activation in the DLPFC/FEF cortex of MS patients and the FPC/FEF cortex of HC was stronger during backward walking (33). Stratified analysis of MS patients based on disability status using the Expanded Disability Status Scale (EDSS, ≥4) and Patient Determined Disease Steps (PDSS, ≥3) revealed that PFC activation levels were higher in MS patients with severe disability (26, 29).

### 3.5 Cortical activation under dual-task conditions

#### 3.5.1 Dual-task vs. single motor tasks

Compared with single motor tasks, dual-task conditions further alter cortical activation levels, with distinct patterns observed between patients with MS and HC. 3 studies showed that during dual-task performance, HC exhibited greater activation in the PFC and its subregions (DLPFC/rPMC/FPC/FEF), as well as faster gait speed, compared with MS patients (10, 24, 33). 4 other studies indicated that MS patients generally walked slower and showed stronger PFC activation than HC under dual-task conditions (29–31, 34). Some studies also reported no significant cortical activation changes in MS patients, despite poorer gait performance relative to healthy individuals (35).

Regarding MS disease subtypes, compared with relapsing-remitting MS patients, progressive MS patients exhibited reduced gait speed and increased PFC activation under dual-task conditions (36, 37). For relapsing MS patients, however, greater activation in the PFC and SMA in response to reduced gait speed was associated with a lower risk of falls (10, 36). In addition, MS patients with higher disability status showed stronger PFC cortical activation under dual-task conditions than those with lower disability status (26).

#### 3.5.2 Dual-task vs. single cognitive tasks (24)

Compared with single cognitive tasks, one study found that activation in the FPA increased as cognitive performance declined in healthy individuals (28). Most studies have shown that compared with HC, patients with MS generally exhibit higher PFC activation but poorer cognitive performance under dual-task conditions (10, 24, 29, 34). 2 studies indicated that cortical activation remained stable in MS patients even when cognitive performance declined (28, 35). However, 1 study also reported that as the complexity of dual cognitive tasks increased, PFC cortical activation decreased in MS patients compared with healthy controls (38).

2 studies showed that, compared with relapsing MS patients, progressive MS patients exhibited increased PFC activation but poorer cognitive performance (lower number of correct letters) under dual-task conditions (36, 37). In addition, compared with single cognitive tasks, MS patients with high disability status showed higher PFC activation levels and poorer cognitive performance under dual-task conditions than those with low disability status (26).

#### 3.5.3 Task load

Compared with single cognitive tasks, as the complexity of dual cognitive tasks increased, PFC activation decreased under dual-task conditions in patients with MS relative to HC (38). When walking while crossing predictable/unpredictable obstacles, MS patients showed higher activation in the PMC, MC, and somatosensory association cortex (SAC) compared with HC. During walking with unpredictable obstacles, MS patients exhibited increased attentional demands and cognitive load, accompanied by more widespread reductions in cortical activation (39). An assessment of attentional demands during dual tasks using partial body weight support to reduce individual burden in MS patients revealed that poorer cognitive performance was associated with enhanced PFC cortical activation, compared with conditions without body weight support (31).

#### 3.5.4 Virtual reality vs. real-world tasks

1 upper limb motor study used a combined virtual and real-world approach to assess cortical activation. In both VR and real-world single motor tasks, patients with MS showed lower total hemoglobin concentration and weaker activation in the PMC and SMA compared with HC, indicating reduced neurovascular activation in MS patients. Under VR dual-task conditions, PMC and SMA activation in MS patients was higher than in healthy individuals. No significant differences in cortical activation were observed in MS patients across the four task conditions (VR and real-world single/dual tasks), suggesting impaired neurovascular adaptability in this population (40).

### 3.6 Effects of MS subtype and disability Status on cortical activation

Disease subtype in patients with MS profoundly affects the efficiency of neural compensation in the brain. Patients with relapsing-remitting MS compensate by increasing PFC activation to maintain gait stability and reduce fall risk. In contrast, patients with progressive MS exhibit increased cortical activation but poorer motor and cognitive performance, indicating excessive recruitment of additional brain resources, impaired neural compensation, and reduced plasticity. Concurrently, MS patients show a consistent pattern across single- and dual-task conditions: higher disability severity is associated with greater cortical activation. This suggests that individuals with low disability can flexibly modulate brain activation to adapt to task demands, reflecting preserved neural regulation capacity. In contrast, patients with high disability must engage more neural resources to meet complex task conditions, resulting in stronger cortical activation but poorer performance, indicative of reduced neural efficiency. Notably, MS disease subtype and disability status often coexist. For patients with progressive MS and severe disability, cortical compensation imbalance may be particularly pronounced, potentially manifesting as overload compensation or decompensation.

### 3.7 Effects of cognitive fatigue and task load on cortical activation

Cognitive fatigue disrupts PFC hemodynamic responses and functional connectivity, leading to impaired neurovascular coupling. As task load increases (e.g., dual tasks, unpredictable obstacle avoidance, backward walking) and individual fatigue levels rise, cortical activation patterns in patients with MS become further disorganized. Some MS patients even exhibit reduced cortical activation levels, indicating a decline in neural compensatory mechanisms.

### 3.8 Effects of interventions on cortical activation

Compared with conventional overground training, robot-assisted gait training can significantly reduce excessive activation of the motor cortex and improve neural resource utilization efficiency in MS patients. Partial body weight support effectively reduces cognitive load during dual tasks, alleviating neural compensatory pressure in patients with high disability. VR and augmented reality (AR) induce similar hemodynamic responses, yet they prominently reveal impaired neurovascular adaptability in MS patients.

## 4 Discussion

This is the systematic review to analyze cortical hemodynamic characteristics in patients with MS under single/dual-task conditions using fNIRS. Overall, MS patients showed a tendency toward increased cortical activation across multiple measured brain regions (e.g., FPC, PMC, SMA, SMC) under both task conditions, with greater activation typically observed during dual tasks. However, these patterns were not consistent across all studies: some reported stronger activation in HC, or no significant changes in activation despite MS poorer performance, or reduced activation in specific regions as task complexity increased. Notably, this apparent hyperactivation occurred alongside poorer motor and cognitive performance, suggesting potential compensatory mechanisms. The heterogeneity in findings, related to cortical regions, task characteristics, population diversity, disability status, and disease stage, aligns with previous studies (41).

### 4.1 Possible explanations for cortical activation

The cortical activation patterns observed in this review may be closely related to the connectivity, efficiency, and interactions between cortical networks. In patients with MS, inflammatory demyelination, axonal damage, and cortical lesions in the central nervous system disrupt structural connectivity between the motor network, cognitive control network, and salience network, reducing the efficiency of information transfer across brain networks (42). Based on the compensatory redistribution model, the brain recruits additional cortical resources for functional reorganization to maintain normal behavioral performance (43). However, this reorganization is not uniform but shows clear task-specific characteristics. For example, in studies involving walking combined with reciting or mental arithmetic, activation in the prefrontal cortex (especially the DLPFC and FPC) is more prominent, as this region is involved in multiple functions such as working memory, motor planning, decision-making, and the integration of cognition and movement (44). In studies involving spatial judgment tasks, the PMC, SMA, and SMC show activation advantages, as these regions are involved in sensorimotor integration, primarily responsible for motor planning, selection, and integration of sensory input and motor output (45, 46). This difference in activation patterns reflects the dynamic allocation of brain resources based on task demands, representing interactions between cortical regions. These findings are consistent with previous studies of neurological disorders using fNIRS and fMRI, which reported significant PFC activation in older adults and healthy young individuals during obstacle walking, serial subtraction, and letter generation tasks (22, 47). Additionally, MS patients exhibit extensive white matter tract damage and thinning of the prefrontal cortex and superior frontal gyrus (SFG) (48). During walking tasks, the brain requires additional cognitive resources, such as integrating multisensory information, implementing purposeful movements, and adjusting posture. Despite the diffuse damage in the prefrontal regions of MS patients, compensatory recruitment is maintained to support adequate task performance. However, as task complexity increases, the demand for brain resources may exceed available capacity, ultimately leading to functional decompensation, manifested as increased cortical activation but deteriorated behavioral performance (49).

Under normal conditions, the human body performs automated motor control via the M1, cerebellar motor areas, and spinal circuits, requiring only minimal activation of the PFC to complete tasks without sustained attentional monitoring or executive control (50). In patients with MS, however, these pathways for automated control are damaged. Consequently, indirect pathways projecting from the PFC and SMA to the basal ganglia, thalamus, and midbrain motor regions become activated, forcing reliance on top-down cognitive control by higher-order cortical areas. This results in increased cortical activation levels (51). Under single-task conditions, this compensation can partially support motor and cognitive performance, yet the increased activation indicates greater neural resource consumption and reduced information processing efficiency, suggesting that the patients' cerebral functional reserve has been excessively recruited (52). Under dual-task conditions, cortical activation is further augmented. For MS patients, impaired automatic control and compensatory reliance on executive control may lead to excessive competition for limited executive resources, resulting in declines in both motor and cognitive performance—a phenomenon widely recognized as a marker of reduced neural efficiency (53, 54).

Elevated attentional load is a key factor exacerbating abnormal cortical activation. The inability to effectively allocate attentional resources to motor and cognitive tasks, regardless of whether patients have MS or other neurological conditions, is a critical cause of reduced motor and cognitive performance during dual tasks (55–57). Posner et al. conceptualized attention as an anatomical network whose primary function is to modulate the operation of other brain networks (58). In MS patients, damage to the attentional network leads to overload in attentional demands and impaired allocation processing during dual tasks, which often results in increased competition and information processing interference in the brain. This forces the cortex to increase both the intensity and scope of activation, yet task performance remains uncompensated (59). This aligns with previous studies showing that both patients with neurological disorders and healthy individuals exhibit poorer cognitive and walking performance when attentional resources are divided between multiple tasks under dual-task conditions (60, 61). Thus, impaired attentional capacity or insufficient resource allocation may act synergistically with reduced neural efficiency, manifesting as the dual-task “cost” characterized by high cortical activation accompanied by low behavioral performance (62, 63).

Notably, changes in cortical activation in MS patients must be interpreted in the context of NVC theory. NVC refers to the dynamic relationship between neuronal activity and cerebral blood flow, where increased neuronal activity leads to a corresponding rise in local cerebral blood flow to meet metabolic demand, a process reflected as an increase in oxygenated hemoglobin signals in fNIRS (64). Under dual-task conditions, MS patients must simultaneously process motor and cognitive information, significantly increasing cerebral metabolic demand. To cope with this load, activation in the PFC (particularly the DLPFC) is enhanced to support postural control and working memory load, consistent with previous findings (65, 66). Furthermore, dynamic changes in neuroplasticity and functional reorganization influence the compensatory efficiency of NVC. In the early stages of MS, neuroplasticity can maintain basic function by recruiting additional cortical resources. However, as the disease progresses, this compensatory capacity gradually diminishes, and patients can only compensate for functional loss by further increasing brain activation, ultimately forming a maladaptive activation pattern (67–69). Previous studies have shown that older adults with mild disability and those with progressive multiple sclerosis often recruit more neural resources even during relatively simple tasks (70, 71). In addition, chronic inflammatory responses and demyelinating lesions in MS can directly damage vascular endothelial function, leading to abnormal platelet adhesion, reduced cerebral vascular autoregulation, and further disrupting the matching between neural activity and blood flow (72, 73). Under the high metabolic demand of dual tasks, the brain cannot effectively regulate blood flow supply to meet neuronal activity requirements, exacerbating cognitive and motor dysfunction (74). In summary, the combined effects of dynamic neuroplastic changes, inflammatory responses, and vascular dysfunction disrupt neurovascular coupling balance in MS patients, resulting in the paradoxical phenomenon of abnormal cortical activation alongside functional decline under dual-task conditions.

### 4.2 Clinical implications

The findings of this study provide guidance for disease assessment and rehabilitation interventions in patients with MS. First, cortical activation levels and oxygenated hemoglobin metabolic characteristics measured by fNIRS can be used as objective evaluation indicators. Combined with activation differences between single- and dual-task conditions, these data can help assess patients' neural functional status, identify the risk of cerebral compensation overload, and assist in evaluating disease severity and rehabilitation potential. Second, considering the characteristics of reduced neural efficiency and poor dual-task load tolerance in MS patients, a stepped dual-task rehabilitation training program can be implemented to gradually optimize cortical activation patterns, improve neuroplasticity, and enhance task performance.

### 4.3 Limitations

This review has several limitations. First, most included studies had small sample sizes and were cross-sectional in design. Large-scale cohort or longitudinal studies are needed to improve the generalizability of the conclusions. Second, participants' ages ranged from approximately 30 to 75 years, with diverse task types and varying disease progression, which may have contributed to result heterogeneity. Third, confounding factors were not well controlled in the included studies, potentially affecting both cortical activation levels and behavioral performance. Additionally, the fNIRS methodology used in this research has inherent limitations, particularly prominent in dynamic scenarios such as walking and dual tasks. Walking activities are prone to introducing motion artifacts, and physiological signals can be easily confused with cortical activation signals. The limited detection depth of fNIRS devices only allows coverage of superficial cortical layers. Furthermore, calibration standards for short-distance reference channels are inconsistent across studies, affecting the comparability of results. Finally, this study employed only a single neuroimaging modality, making it difficult to simultaneously integrate neural electrical activity information, which imposes limitations on the interpretation of brain function.

### 4.4 Future research perspectives

Advances in multimodal neuroimaging techniques have made the combined use of EEG and fNIRS an important direction in mobile neuroimaging research. fNIRS and EEG exhibit significant complementarity in such studies: fNIRS provides good spatial resolution, reflects cortical hemodynamic changes, and is more tolerant to motion artifacts, making it particularly suitable for dynamic tasks such as walking (75). In contrast, EEG captures neural electrical activity with millisecond-level temporal resolution, revealing the dynamic temporal characteristics of neural activity (76). Combined multimodal neuroimaging can simultaneously achieve high temporal and spatial resolution monitoring of neural activity, offering a more comprehensive perspective for understanding the relationship between neural activity and hemodynamic coupling in MS patients during mobile scenarios. Meanwhile, building on the limitations of the current study, future research can advance in several directions: First, Conduct large-sample, multi-center prospective data studies using unified task paradigms and data processing standards. Second, Implement study grouping with strict control of confounding factors such as disease subtype, disability severity, and cognitive status. Third, Actively carry out interventional trials to explore the effects of rehabilitation training and clinical medications on improving cortical activation patterns and behavioral abilities, thereby tapping into the application value of fNIRS in therapeutic efficacy evaluation.

## 5 Conclusions

Certain MS patients demonstrate increased task-related cortical activation accompanied by reduced performance. While this may reflect alterations in neural efficiency or neurovascular coupling, it could also indicate compensatory recruitment or increased attentional effort, depending on the task, disability severity, and disease-related factors. fNIRS may serve as a useful monitoring tool, and future multimodal EEG-fNIRS research is warranted to clarify the underlying mechanisms.

## Data Availability

The datasets presented in this study can be found in online repositories. The names of the repository/repositories and accession number(s) can be found in the article/Supplementary Material.
