# Correction: The relationship between motor and cognitive performance and cortical activity in patients with multiple sclerosis: a systematic review of functional nearinfrared spectroscopy studies

**DOI:** 10.3389/fresc.2026.1941006

**Published:** 2026-08-31

**Authors:** Hongjiao Sun, Haonan Xing, Kechun Hu, Zhongyu Wu, Jiawei Wu

**Affiliations:** 1 Department of Inpatient and Medical Record Management, Air Force Medical Center, Air Force Medical University, PLA, Beijing, China; 2 Rehabilitation Hospital Affiliated to National Research Center for Rehabilitation Technical Aids, Beijing, China

**Keywords:** brain, dual-task paradigm, fNIRS, motor-cognitive performance, multiple sclerosis

Affiliation “Rehabilitation Hospital Affiliated to National Research Center for Rehabilitation Technical Aids, Beijing, China” was omitted for author Haonan Xing.

This affiliation has now been added for author Haonan Xing.

Author Haonan Xing was erroneously assigned to affiliation “Department of Neurological Rehabilitation, China Rehabilitation Research Center, Beijing, China”.

This affiliation has now been removed for author Haonan Xing.

Authors Hongjiao Sun and Haonan Xing were erroneously omitted as equal contributing authors.

The original version of this article has been updated. 

